# SPACER: server for predicting allosteric communication and effects of regulation

**DOI:** 10.1093/nar/gkt460

**Published:** 2013-05-21

**Authors:** Alexander Goncearenco, Simon Mitternacht, Taipang Yong, Birgit Eisenhaber, Frank Eisenhaber, Igor N. Berezovsky

**Affiliations:** ^1^Computational Biology Unit and Department of Informatics, University of Bergen, Bergen 5020, Norway, ^2^University Library, University of Bergen, Bergen 5020, Norway, ^3^Bioinformatics Institute (BII), Agency for Science, Technology and Research (A*STAR), 30 Biopolis Street, #07-01, Matrix, 138671, Singapore, ^4^Department of Biological Sciences (DBS), National University of Singapore (NUS), 8 Medical Drive, 117597, Singapore, ^5^School of Computer Engineering (SCE), Nanyang Technological University (NTU), 50 Nanyang Drive, 637553, Singapore and ^6^Department of Biological Chemistry, Weizmann Institute of Science, Rehovot 76100, Israel

## Abstract

The SPACER server provides an interactive framework for exploring allosteric communication in proteins with different sizes, degrees of oligomerization and function. SPACER uses recently developed theoretical concepts based on the thermodynamic view of allostery. It proposes easily tractable and meaningful measures that allow users to analyze the effect of ligand binding on the intrinsic protein dynamics. The server shows potential allosteric sites and allows users to explore communication between the regulatory and functional sites. It is possible to explore, for instance, potential effector binding sites in a given structure as targets for allosteric drugs. As input, the server only requires a single structure. The server is freely available at http://allostery.bii.a-star.edu.sg/.

## INTRODUCTION

Protein function depends on the inherent dynamics of the protein structure. Not only is the balance between different conformational states of importance in this context, but also how easily the transitions between them occur. The external factors, such as ligand binding or local chemical modifications, can affect the conformational ensemble and shift the equilibrium toward (in)active conformations. The regulation is called allosteric when the effector site is not directly adjacent to the site of altered activity (1). The early phenomenological Monod-Wyman-Changeux (MWC) (2) and Koshland-Némethy-Filmer (KNF) (3) models were devised to explain a classic example of allosteric regulation (4): the cooperative ligand binding of many oligomeric proteins, where binding of substrate to one subunit affects the ligand affinity in other identical subunits. The MWC model postulates that binding stabilizes one of several available conformations with emphasis on symmetry conservation, whereas the KNF model assumes an induced-fit scenario. Since the MWC and KNF models, numerous studies have been performed at different levels of coarse-graining (5). The models themselves have been expanded as well, and allostery is currently considered in proteins of different size, shape and degree of oligomerization, spanning from small single-domain structures to the large chaperones (6,7). Originally, there was an apparent dichotomy between MWC and KNF models and their counterparts in the energy landscape-based ‘new view’ of allostery (8–11)—conformational selection and induced fit. The main difference between the two models is whether binding precedes conformational change (11). Transition pathway analysis is primarily a matter of kinetics, whereas the shift in conformational equilibrium is one of thermodynamics: the conformational states involved determine which binding sites are allosterically connected, and their relative stability before and after binding determines the effect of regulation (12). Overall, the two models do, however, not describe mutually exclusive scenarios (6,11): in both cases, there is a shift in the population of different functional states on effector binding. The issue was resolved with the introduction of a more general physical framework (13).

Despite the progress achieved in the understanding of allostery, most of studies have been performed on individual proteins or small collections of them (6,7,14). The previously developed approaches to the analysis of protein dynamics are mostly focused around the analysis of the energetics of the protein’s structural ensemble, mobility of individual residues and conformational changes. For example, the COREX/BEST algorithm (15) enumerates the protein ensemble, defines the relative free energies of each state and characterizes the energetics of the ensemble. The AD-ENM server performs an analysis of macromolecular dynamics based on the calculation of the spectrum of normal modes for the elastic network model (16). The ProDy project allows to analyze dynamical properties of individual residues and to visualize protein dynamics (17). However, a general molecular description of allosteric regulation that allows prediction of allosteric sites based on protein dynamics, and that explains molecular mechanisms of communication between sites was still lacking (5). Resorting to the thermodynamic view of allostery (5–7), we developed the concepts of binding leverage and leverage coupling that allow quantifying (i) the coupling between ligand binding and the intrinsic dynamics of the protein and (ii) the communication between different binding sites. These concepts also allow finding latent effector binding sites, which along with known ones can be considered as potential targets for allosteric drugs (5).

In the era of structural proteomics, with an exploding number of protein structures, it is of crucial importance to have instruments that allow massive and efficient analysis of multiple protein targets. For studying allostery, there are several important requirements for such an instrument. It should be based on a generic molecular model of allostery, which works regardless of the size, degree of oligomerization or function of the protein. It should work with a single structure, regardless of it corresponds to the active/activated or inactive/inactivated state of the protein. It should be able to explore communication between natural allosteric and catalytic sites, to detect latent sites in the structure, as well as to analyze sites chosen by the user. The SPACER server satisfies the aforementioned requirements, providing reasonably fast interactive tools for exploratory analysis of allosteric communication. Later in the text, we provide a brief description of the theoretical background for SPACER’s methods followed by a practical guide to exploratory analysis of allosteric communication with SPACER. An online tutorial (http://allostery.bii.a-star.edu.sg/tutorial/) exemplifies the server workflow for the case of the Phosphofructokinase (PFK) homotetramer, showing the major options in the SPACER and explaining the most important features and results.

## THEORETICAL BACKGROUND

The balance between different conformations of a protein and the role of ligand binding in switching between its functional states are the major determinants of allosteric regulation and communication. The steps in the analysis of allostery in a given protein structure should include, therefore: (i) prediction and characterization of the substrate- and effector-binding sites; (ii) characterization of coupling between the ligand binding and functional dynamics to determine regulatory sites; and (iii) analysis of the communication between the allosteric and catalytic sites, both known and latent ones, as well as sites of interest designated by the user. Later in the text is a brief description of the major methods used in SPACER. It includes two instruments for the search of allosteric sites: ‘local closeness’ (14) based on static geometric features of the structure and ‘binding leverage’ (6) based on protein dynamics measures. It also includes ‘leverage coupling’ (7) that quantifies communication between the allosteric and catalytic sites in the protein.

### Local closeness

Local closeness (14) is a geometry-based predictor of ligand-binding sites involved in protein function and regulation. It detects potential allosteric sites that are not necessarily characterized by specific chemical groups (as in the catalytic residues) selected in evolution and manifested in sequence conservation. Local closeness is a local centrality measure. It quantifies residue connectivity to neighbors within a finite distance *m* in the residue interaction graph (RIG), where each residue in the protein is a node. The RIG is built on the van der Waals contacts between atoms (including hydrogen) of the amino acid residues. The local closeness of degree *m* for a node is defined as

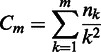

where *n_k_* is the number of nodes whose shortest distance from a given node is exactly *k*. The local closeness of degree four (*m* = 4) gives the best performance of predictions (14). This value of *m* effectively means that only the residues closer than 30–40 Å are included in the calculation, which roughly corresponds to the size scale of single domains. It is recommended, therefore, to use *m* = 4 in most of the calculations. Smaller values of *m* are recommended when only small cavities on the surface are to be investigated.

### Binding leverage

Binding leverage (6) measures the ability of a binding site to couple to the intrinsic motions of a protein by quantifying the cost of the binding site deformation when a ligand is present and is resisting the motion. Potential binding sites are found by a coarse-grained docking procedure [see ‘Implementation’ section and reference (6)]. Conformational changes are approximated here by low frequency C_α_ normal modes. The binding leverage *L_A_* for a set of normal modes *A* is calculated as

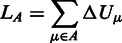



Δ*U_μ_* represents the total change in potential energy of a set of springs owing to the motion of a normal mode *µ*. Springs of length *d_ij_* are placed between all pairs of C_α_ atoms *i* and *j*, whose connecting line passes within 3.5 Å of any ligand atom (*κ* is an arbitrary spring constant), giving the following expression for Δ*U*:

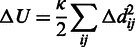



The binding leverage of a site both depends on the range of the motion at the site and how many pairs of residues interact with the ligand. A ligand that binds to a site with high binding leverage has a potential to lock one or more collective degrees of freedom (represented here by normal modes).

### Leverage coupling

We introduced the concept of leverage coupling to provide a quantitative characteristic of allosteric communication (7). It is based on the assumption that sites that have high binding leverage for the same motion are more likely to be allosterically coupled than sites that only have high binding leverage for motion along independent degrees of freedom.

The strength of communication between two sites *P* and *Q* is defined as a dot product of binding leverages, *λ_P_* and *λ_Q_*, of these sites:

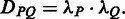



The vector of the binding leverage of site *P* is defined as 
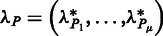
, where 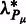 is a binding leverage of the site *P* caused by the normal mode *µ*:

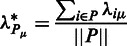

where the norm of *P* is the number of elements in the set.

The normalized leverage coupling *C_PQ_*

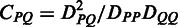



has the range 0 ≤ *C_PQ_* ≤ 1. The normalized leverage coupling *C_PQ_* is necessary for the analysis of big molecular machines like chaperones (7), where the conformational change at the binding sites is small compared with the large-scale functional motions. In this case, the task is to compare the values between different sites and to find the most correlated pairs of the sites for a given protein. The measure *C_PQ_* is thus used (instead of *D_PQ_*) to analyze how binding sites are correlated with different modes of functional motion. As a result, leverage coupling allows one to investigate allosteric communication in enzymes regulated by ligand binding and phosphorylation in proteins with different sizes and degree of oligomerization (7).

## IMPLEMENTATION

The SPACER server is written in Python using the Pyramid framework (http://www.pylonsproject.org/). The server has a modular distributed architecture, where the modules communicating via the Advanced Message Queuing Protocol are asynchronously connected by a Celery distributed task queue (http://www.celeryproject.org/). Docking and binding leverage modules are implemented in C, local closeness in C++. The interactive web interface is powered by the javascript libraries jQuery (http://www.jquery.com/) and d3js (http://d3js.org/), and by the Jmol molecular viewer (http://www.jmol.org/) for visualizing the protein structures. The normal mode analysis is done using C_α_ elastic networks using the Molecular Modeling Toolkit (18). The SPACER server interacts with the Protein Databank (19) and PDBePISA (20) on the fly.

## USING THE SPACER SERVER

The server is designed for interactive exploratory analysis of protein structures. Therefore, there is no special programmatic interface for automatic execution of tasks. However, we provide machine-readable output files for every step of the analysis workflow.

The binding leverage calculations, including normal mode analysis and docking, may take a significant amount of time depending on the size of the protein complex (about an hour on average, up to 40 h for chaperones). To minimize the waiting time, we have pre-calculated many proteins with the default parameters. The session menu in SPACER interface indicates the status of jobs, whether they are ready or still running. There is also a link for restoring the session should the user decide to return to the results later. In general, writing down the session ID (five or six characters) should be sufficient for switching back to the analysis at any time or sharing the results with colleagues. It is only possible to work with one structure at a time in a session. The session is stored for at least 6 months from the moment of last access.

**Figure 1.**
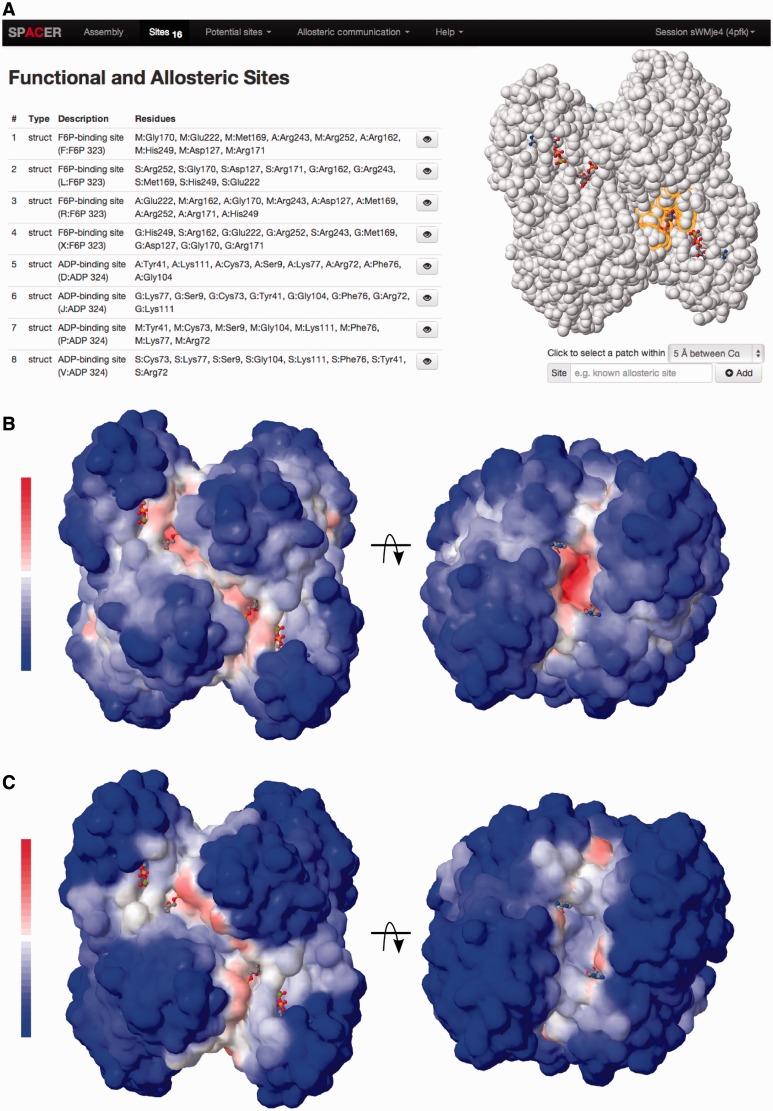
Exploring the effector and catalytic sites. SPACER screenshots: (**a**) showing the list of sites, one selected site, and the tool to add user-selected sites interactively; (**b**) Local closeness tool showing the results in Phosphofruktokinase (PFK, PDB id 4pfk) in two projections; (**c**) Binding leverage tool showing the results in PFK in two projections.

**Figure 2.**
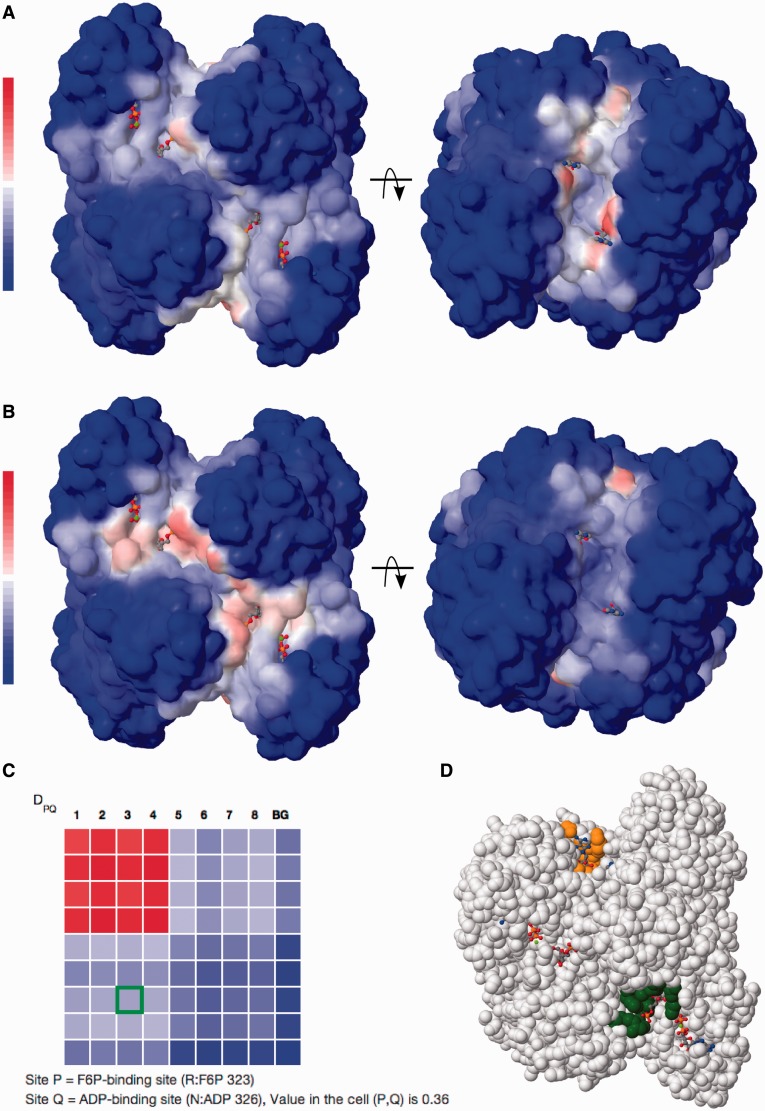
Exploring the allosteric communication between the sites. SPACER screenshots: (**a**) showing the leverage coupling between an ADP-binding (activator) site in PFK and the rest of the structure; (**b**) leverage coupling between F6P binding site and the rest of the structure. (**c**) The DPQ matrix shows communication between eight sites in PFK (four sites in each subunit): F6P-binding sites (1–4) and ADP-binding sites (5–8), the last row and column in the matrix designate communication with the rest of the structure (background, BG). The values are color-coded from blue (0) to red (1) via white (0.5). A pair of allosteric sites (3 and 7) is selected and highlighted with a green border in the matrix. (**d**) Selected pair of sites (orange and green) shown directly on the protein structure.

To exemplify the work and major options provided by the server, we use the tetrameric enzyme PFK, which displays a classic example of allostery. The enzyme is allosterically inhibited by phosphoenolpyruvate and activated by ADP binding to the same site. It is cooperative with respect to binding of the two substrates, fructose-6-phosphate (F6P) in the presence of phosphoenolpyruvate. We use the crystal structure supplemented by the allosteric activator ADP (PDB ID 4pfk). What biological conclusion can the user obtain with the help of SPACER? First, the sites with highest local closeness and binding leverage correspond to known ligand-binding sites in PFK (Figure 1). Second, Figure 2a shows that there is weak communication between the ADP-binding site and F6P-binding active sites. However, the communication between different ADP-binding sites is stronger. Third, Figure 2b shows strong communication between the F6P-binding (functional) sites and weak communication of the functional sites with the ADP-binding (regulatory) sites. Finally, the color-coded matrix *D_PQ_* shows an overview of the levels of pairwise communication between all defined sites (Figure 2c). The values are normalized to the interval from zero (no communication—blue) to one (strongest measured communication—red); white color corresponds to weak communication. The matrix is interactive; the selected pair of sites is highlighted by color in the molecular viewer (Figure 2d). Importantly, the matrix also shows the level of communication between the sites and parts of the structure not belonging to any site (background, abbreviated as BG). The background communication is used as a control and can be subtracted from the values by switching the matrix display mode. The analysis of large protein complexes such as chaperones requires a special normalization (*C_PQ_*), as the values may not be directly comparable between the subunits and within the subunits. The *C_PQ_* matrix can be shown for any structure by activating the respective display mode. Below is a step by step description of the server’s options and the outputs of calculations with explanations of the illustrations and downloadable data.

### Input

The analysis of allosteric communication requires a protein structure representing the biologically relevant assembly—a protein complex in its natural oligomeric configuration. The easiest way to start is to provide a PDB ID. SPACER will then try to find the most probable biological assembly in the PISA and PDB databases. In case of PFK enzyme (PDB ID 4pfk), the best assembly is retrieved from PISA and displayed in the embedded Jmol viewer on the Biological Assembly Page (see tutorial for illustrations). If no assembly is found, the structure will be fetched from the Protein Databank as is. Alternatively, the user can provide the atomic coordinates in PDB format. The only requirement is that the file should have consistent residue/atom naming and numbering, according to the standard PDB format. SPACER will show the assembly structure for visual control with the subunits in different colors. At this stage, it is possible to remove some of the protein chains, in case it is only required to analyze a part of the protein complex (for instance, a subunit of a chaperone). Removing the chains might affect or even significantly change the allosteric communication compared with the picture obtained for the native protein structure.

### The sites

The SPACER server analyzes allosteric communication between sites in protein structures. Therefore, it is essential to identify the sites. If the ligands are present in the PDB file, the corresponding catalytic and effector-binding sites are added automatically. Otherwise, the sites can be added manually. The local closeness and binding leverage tools can help in identifying potential ligand binding and functional sites. We provide an interactive way of analyzing the sites of interest manually by clicking on any atom on the protein surface (Figure 1a). The site will include the residues closely located to the selected one (the radius can be adjusted). It is also possible to edit the list of residues by specifying the chain name and index or each residue. In case of PFK described in the tutorial, the user is provided with the list of four ADP- and four F6P-binding sites extracted from a given PDB structure (Figure 1a). Once the sites are defined, the allosteric communication between them can be explored using leverage coupling.

The ‘local closeness’ tool requires only a single parameter—the degree of the RIG, set by default to four. The results are shown as colored surface, where the highest values (red) correspond to the potential binding sites (Figure 1b). The user can add the sites to the list by clicking on the red patches with high values. The local closeness results can be downloaded as a PDB file with the values stored as B-factors or as a table in plain text (tab-separated csv format). Two orientations of the PFK structure are shown in Figure 1b with surfaces gradually colored from blue to red depending on the value of the local closeness. As a control of recall of known binding sites, we show the averaged values of local closeness alongside the defined sites. Using the values for the known sites as a ground level makes it easier to identify and add the potential binding sites of interest manually.

The ‘Binding leverage’ tool uses Monte Carlo docking simulations to probe the surface of the protein (6). The main parameter is the probe size—the probe is modeled as a peptide with 2–6 C_α_ atoms. The default probe size is four C_α_ atoms. The probe should be small enough to fit the cavities, but large enough to not get buried in sites not accessible to the real ligands. Binding leverage effectively measures the coupling between probe binding and the deformations described by the lowest frequency normal modes. Therefore, the effect of probe size on the calculation results can be significant. After changing the parameter, a recalculation of the probe docking is needed, which might take some time, depending on protein size. Importantly, in the search for ligand-binding sites, the ligands already existing in the structure are excluded. Binding leverage results for PFK are shown as colored surface (Figure 1c) and can be downloaded as plain-text table or a PDB file with B-factors. It is possible to add additional sites of interest while exploring the binding leverage results. As in the case of local closeness, the averaged values of binding leverage are also shown alongside the already listed sites, making it easier to add new sites based on the leverage values.

### Allosteric communication

Exploring the allosteric communication is the final step of SPACER achieved by calculating the leverage coupling. There are two ways of showing leverage coupling: (i) communication between a given site and the rest of the protein structure, (ii) communication between pairs of annotated sites. In the former case (shown for PFK, Figure 2a and b), the results are shown as a colored surface and can be downloaded as a PDB file with B-factors or as a plain-text table. In the latter case (Figure 2c), the results are shown in the form of colored symmetric matrix (*D_PQ_* with options for the background analysis, and *C_PQ_* (optional) for the analysis of big molecular machines, see also theoretical background). Each cell in the matrix corresponds to a pair of sites *P* and *Q*, and the color show the strength of allosteric communication between the sites. The interactive tool will show a pair of communicating sites on the structure once the user clicks on the corresponding cell in the matrix (shown for PFK, Figure 2c and d). The last row and column in the matrices shows the leverage coupling with the rest of the structure (background). The background is defined as the residues not included in any of the described sites. If the background value is high, it may indicate that some potential effector-binding/catalytic sites are not listed and should be added. The resulting matrices can be downloaded in machine-readable JSON format.

## CONCLUSIONS AND OUTLOOK

The SPACER server establishes an interactive exploratory framework for finding allosteric and catalytic ligand-binding sites and for analyzing the communication between them. SPACER implements a unique approach that provides simple and meaningful physics-based quantities for characterizing the link between structural dynamics and binding of effector molecules. It is applicable to a wide range of proteins—from small monomeric structures to large protein complexes (6,7). Importantly, it works with a single crystal structure, which is sufficient for representing the conformational ensemble of the protein (6).

The results of the analysis provided by SPACER can be used to study many different areas, such as protein function, regulation and evolution of protein function, protein dynamics, X-ray and nuclear magnetic resonance analysis, drug design and so forth. In particular, the presented approach allows one to detect latent allosteric sites or analyze user-selected sites of interest, which can be further explored as potential targets for allosteric drugs (5,21).

## FUNDING

Funding for open access charge: Functional Genomics (FUGE II) Program, Norwegian Research Council.

*Conflict of interest statement*. None declared.
